# When Should Surgical Cytoreduction in Advanced Ovarian Cancer Take Place?

**DOI:** 10.1155/2010/852028

**Published:** 2009-10-25

**Authors:** Igor E. Martinek, Sean Kehoe

**Affiliations:** Oxford Gynaecological Cancer Centre Surgery and Diagnostics, Level 0, Churchill Hospital, Oxford OX3 7JL, UK

## Abstract

Initial surgical management is commonly accepted to date as paramount in the treatment of women presenting with epithelial ovarian cancer and permits the assessment of the disease (staging), the histological confirmation of disease type and grade, and the practice of maximal debulking preceding platinum-based chemotherapy. Many studies have shown that the volume of residual disease after initial surgical cytoreduction inversely correlates with survival. Thus, women with optimal debulking performed by a trained specialist have improved median survival. In this review, we will focus on the answers gleaned from clinical trials on primary and interval surgery, which prompts the question on the timing of surgery in respect to chemotherapy. Interval debulking surgery (IDS) is secondary cytoreduction following primary debulking and is carried out in between the courses of chemotherapy. The major clinical trials and the latest systematic reviews seem unable to give any definitive guidance or recommendation for clinical practice. The choice of aggressive primary cytoreduction or upfront chemotherapy followed by second line surgical cytoreduction seems among others to have to be individualized according to tumour load, prediction of its resectability, and response to chemotherapy. The role of tumour biology must also be kept in mind. Finally, concrete answers are awaited on the timing of surgery from the ongoing prospective randomized control trials (CHORUS and EORTC 55971) though preliminary data from the latter have already been presented at major meetings (IGCS 2008; SGO 2009) and ignited strong debate.

## 1. Introduction

Ovarian cancer represents the sixth most commonly diagnosed cancer among women in the world and causes more deaths per year than any other cancer of the female reproductive system [1]. In advanced disease which constitutes about 75% of women at presentation, the accepted management is a combination of surgery and platinum based chemotherapy. This has been the approach for some decades, though the 5-year survival remains poor at about 40%. Epithelial ovarian cancer constitutes the majority of disease types, and this review will focus on reports relating to advanced epithelial ovarian carcinoma.

## 2. Materials and Methods

A Medline database search (January 1966 to April 2009) was undertaken using key words: epithelial ovarian cancer, debulking surgery, and interval debulking surgery resulting in 80 articles with 14 relevant papers. The articles in full were obtained for each of the papers and reviewed by the authors. Results in terms of overall survival (OS) and progression free survival (PFS) were evaluated in each study.

## 3. Results

The 80 resulting articles were screened and 14 relevant papers were retained: 3 meta-analysis [2–4], 3 randomized control trials (RTC) [5–7] (Table 1) 2 Cochrane Reviews (CRs) [8, 9], and 6 case/control (CC) reports enrolling more than 50 patients [10–15] (Table 2).

## 4. Discussion

### 4.1. Primary Debulking Surgery

The initial studies supporting the concept of debulking surgery were published in the 1970s by Griffiths et al. [16]. The premise for considering the potential impact of reducing intra-abdominal tumour burden was based on the findings of work by Magrath et al. [17], which reported enhanced survival outcome by reducing intra-abdominal disease, in patients with Hodgkin's disease. Griffiths undertook a retrospective analysis of just over 100 women and noted that those with residual disease masses <1.6 cms in largest diameter had an improved survival outcome compared with patients left with a greater disease volume. A subsequent small prospective study [18] on a heterogeneous population of patients, who underwent aggressive radical surgery, also revealed the better survival pattern associated with less tumour burden. Thus, the concept of debulking surgery in ovarian cancer became the normal approach to this disease. The use of adjuvant chemotherapy, which is platinum based, is also the accepted norm in care. The question as to whether the surgical ability of the operator or the inherent tumour biology of the disease is the main factor impacting on survival remains a debate. Indeed, the benefit of radical debulking has already come under criticism [19] while some have advocated that tumour biology rather than the surgical effort might determine prognosis [20]. In a study of 213 patients with Stage IIIC epithelial ovarian cancer who underwent complete cytoreduction before initiation of systemic platinum-based combination chemotherapy, Eisenkop and Spirtos [21] came to the conclusion that the need to remove a large number of peritoneal implants correlates with biological aggressiveness and diminished survival, but not significantly enough to preclude long-term survival or justify abbreviation of the operative effort.

Regarding primary surgery, there is a plethora of published papers, all of which support the findings of Griffiths, though none are randomized controlled trials, and hence, all with similar inherent biases. It is also important to note that various definitions of optimal cytoreduction have been proposed [22–24]. The Gynaecologic Oncology Group (GOG) currently defines optimal cytoreduction as leaving residual disease less than 1 cm in maximum tumour diameter. Some may argue that optimum should only mean no macroscopic residual disease.

There are 3 systematic reviews on residual disease and outcome, which have conflicting conclusions. In an analysis of 81 cohorts of patients (over 6000 women) with advanced-stage ovarian carcinoma treated with platinum-based chemotherapy Bristow et al. [4] found a 5.5-percent increase in median survival for every 10-percent increase in the proportion of patients achieving maximal cytoreduction. Contrary to these findings was the meta-analysis by Hunter et al. [2], (again over 6000 women) whereby the administration of platinum was deemed more important in influencing survival rather than the achievement of optimum debulking surgery. The main difference between these papers is that in Bristow's study, all patients were exposed to adjuvant platinum therapy, which was not the case in Hunters study. The third and smaller study also concluded that optimum debulking was associated with improved survival patterns, though further prospective trials were necessary [3].

### 4.2. Secondary Surgical Cytoreduction

At the beginning of the eighties, Berek et al. [25] noticed that secondary cytoreduction could also improve survival. Subsequently, the role of interval debulking surgery (IDS) has been investigated in three prospective Randomized Controlled Trials (RCTs) [5–7] where conclusions are different. Interval debulking surgery is defined as a second operation performed after 3 or 4 cycles of platinum chemotherapy in woman who had suboptimal debulking primary surgery. Table 1 summarizes the main features of these trials.

The trials by Redman et al. [7] and the GOG by Rose et al. [6] failed to show any advantage of IDS. The study by Redman was closed prematurely, as no survival benefit was noted at interim analysis, and of note, optimum debulking was defined as <2 cms residium compared with <1 cms in the other studies. In the GOG study, 550 women with suboptimally debulked stage III/IV ovarian cancer received three cycles of paclitaxel/cisplatin and then were randomly assigned to interval cytoreduction or no surgery. Chemotherapy was continued up to a maximum of 6 cycles. A secondary attempt at cytoreduction was not associated with an improvement in progression free survival (PFS) (12.5 versus 12.7 months) or overall survival (OS) (36.2 versus 35.7 months). This was not the case with the EORTC trial carried out by Van de Burg et al. [5], which showed that the IDS group had a significantly increased median survival of 6 months compared to those who had not undergone this procedure. Indeed this is still the only prospective RCT showing a survival benefit with “debulking” surgery. Nevertheless, it is important to point out some differences between these trials. At the time of the EORTC trial, chemotherapy consisted of cisplatin/cyclophosphamide as Paclitaxel was not available, unlike the GOG trial. Another major difference was that in the EORTC trial, primary surgery was not necessarily performed by a trained gynaecological oncologist, resulting in different extents of debulking. The number of patients with less than 5 cm of residual tumour following primary cytoreduction in the EORTC trial was less than a third, compared to 55 percent in the GOG trial. Surgery performed by a trained gynaecological oncologist has been shown to increase survival [26], and the GOG study therefore concludes that with appropriate persons undertaking primary surgery, IDS is not required.

### 4.3. Neoadjuvant Chemotherapy (NACT) and Debulking Surgery

The term IDS should be confined to patients who have had primary surgical debulking, but it has been used in situations whereby a primary surgical attempt is delayed until during chemotherapy. Six large case-control studies [10–15] relating to “delayed” primary surgery were identified, and are summarized in Table 2.

One of the studies [10, Colombo et al.] divided patients into 2 groups to evaluate the place of surgery in the therapeutic sequence of care: group 1 receiving upfront surgery and group 2 where first debulking was undertaken after chemotherapy. In group 1 the OS was 38 months and 3 factors significantly predicted suboptimal upfront surgery: poor performance status, extensive mesenteric involvemen, and stage IV disease. The second group showed OS of 26 months, and despite a response to NACT in 90% of cases, there was no long-term survivors in the patients whose interval cytoreduction was suboptimal. Generally, OS was stated to be influenced by three main factors: the extent of the disease at the time of diagnosis, the biology of the tumour, and its chemosensitivity, and the authors concluded that optimal surgery with limited morbidity (14% in their case) can be achieved in many cases at primary surgery setting. Hegazy et al. [12] found, in a population of patients with advanced ovarian carcinoma where resectability was not possible, that neoadjuvant chemotherapy* helped to select patients for feasible and relatively less aggressive* IDS, thus preventing initial surgical failure, in terms of optimal debulking. However, Morris et al. [27] in 1989 demonstrated that patients resistant to chemotherapy during primary treatment had little benefit from IDS. This was also concluded by Rafii et al. [14] as well as the selection effect of NACT for the second intention surgery.

In another recent study [13], the complete response rates after three cycles of platinum/taxane chemotherapy was 36.1%. After IDS, 80% of all patients were left with optimal residuals (<2 cms). The response rate to chemotherapy given in a neoadjuvant setting was comparable to those published in literature in patients who were treated with conventional upfront tumour reduction surgery followed by adjuvant chemotherapy. They also found that residual decease after IDS is the only significant predictive factor associated with prolonged PFS (*P* = .003). To date, there is very little good quality evidence to either support or refute the use of neoadjuvant chemotherapy in the treatment of ovarian cancer [9].

A retrospective study between 1980 and 1997 from Vergote et al. [15] included 285 patients with stages III and IV ovarian cancer. In the period from 1980 to 1988, optimal primary cytoreduction (0.5 cm residual disease) was achieved in 82% of cases, but patients with stage IV disease or a metastatic tumour load of >1 kg prior to this procedure had poorer survival with high postoperative mortality (6%). Between 1989 and 1997 patients received either upfront surgery or chemotherapy depending on the extent of the disease and the performance status. This subsequent management improved overall survival, despite a reduction of 25% in the rate of primary debulking.

### 4.4. Surgery at Relapsed Disease

A large Norwegian retrospective study (*n* = 789) [11] carried out at the Radium Hospital looked at treatment model for 1st relapse of ovarian cancer of any stage. They found that treatment free interval (TFI) following primary therapy is a significant prognostic factor for OS in multivariate analysis. They also report age as prognostic factor for OS at the time of secondary cytoreductive surgery. Survival benefit was clear for patients with optimum secondary cytoreductive surgery followed by chemotherapy compared with chemotherapy alone at the time of recurrence. Complete secondary cytoreductive surgery was found possible in a significant percentage of patients properly selected for this secondary surgery. Localised tumour was found to be a significant factor to predict this optimum surgery. This selection of patients for secondary cytoreductive surgery is thus crucial. Guidelines at relapse [11] for local and disseminated disease have been set up, where secondary cytoreductive surgery is recommended as independent of TFI for localized tumours and should be considered for TFI > 24 months in case of disseminated disease (Table 3).

Selecting the right patients for the right treatment sequence is challenging. Predicting the possibility to perform successful surgery has been studied [28, 29] with one model having an 85% specificity or ability to identify patients undergoing optimal surgery [30]. In certain situations laparoscopy is recommended as the most valuable tool for evaluating the operability in upfront or second line debulking surgery [31].

## 5. Conclusions

This paper has reviewed only RCTs and large series, which do reflect the findings of many other reports on the specific debates surrounding the role and timing of surgery in ovarian carcinoma. There is agreement that one of the most important prognostic factors for survival in the treatment of ovarian cancer is the amount of residual tumour after cytoreduction [4, 16]. It is welcome to note that in more recent times surgical approaches have undergone scrutiny in RCTs. Indeed there is evidence of a shift from debulking for all to debulking for a select group, or put another way increased individualisation of therapy. Unlike in previous decades the use of neoadjuvant chemotherapy seems to have gained some popularity, though the real impact requires the formal publication of the randomized trials EORTC 55971 and CHORUS. The EORTC study has been presented at the IGCS in Bangkok and generated a lot of debate, as to the role of neoadjuvant chemotherapy. The finalised peer-reviewed publication is awaited with interest.

Another factor which cannot be ignored in the debate is the inherent tumour biology where the question, raised by some [32] and still requiring an answer, is to know if it is the surgeon's skills or tumour biology which determines survival outcome. In this respect, opinions vary regarding its impact on the ability to surgically debulk [21]. On the other hand, others have put forward the strong expression of the p53 tumour suppressor gene correlating with reduced likelihood of achieving complete cytoreduction [33]. The progress and accessibility to novel technologies applied to biology will make possible in the future the assessment of new prognostic profilesbased on genetic and/or proteomic tumour characteristics. The future also relies on the identification of predictive factors of response to treatment [34].

## Figures and Tables

**Table 1:** RCTs investigating the role of IDS.

Name of Study & year	Rose PG et al. [6] (GOG)	Van der Burg et al. [5] (EORTC)	Redman et al. 1994^1^ [7]
N	550 with 448 randomized 226 IDS versus 222 no IDS	425 with 319 randomized 278 evaluated: 138 IDS versus 140 no IDS	86 randomized with 7 excluded*37 IDS42 no IDS
FIGO stage	II-IV	IIB-IV	II-IV
Trial characteristics	RD > 1 cm after primary surgery and responding/stable after 3 cycles of Cisplatin/pacitaxelStage IV only pleural effusion	RD > 1 cm & maximum primary debulking not attempted in all cases with high proportion of RD >5 cm Randomization after 3 cycles of CP	Primary surgery and RD > 2 cm 1–4 CP Or 3 PAB followed by 5* escalating CP
PFS for IDS versus No IDS	12.5 versus 12.7	18 versus 13	
OS for IDS versus No IDS	36.2 versus 35.7	26 versus 20	15 versus 12 months

CP: cisplatin/cyclophosphamide; overall survival in months; PAB: cyclophosphamide/doxorubicine/bleomycin; PSF: progression free survival in months; RD: residual disease. *Surgery was non suboptimum. ^¹^Randomization begins at the start of the trial.

**Table 2:** Nonrandomized case control studies evaluating delayed primary debulking surgery.

Name of Study	Colombo et al. [10]	Oksefjell et al. [11]	Hegazy et al. [12]	Le T et al. [13]	Rafii et al. [14]	Vergote [15]
N	203	789(217 IDS 572 non IDS)	59 all submitted to prior surgical exploration	61	109	285

FIGO stage	IIc-IV	All stages treated for 1st relapse	II-IV	IV without bowel obstruction	IV	III-IV

Important study data	Gr 1 conventional OS = 38 m Gr 2 with NACT OS = 26 m	Platinum single or combination/taxol single or combinationor other	*N = 27 (*OS* = 25 m) unresectable NACT with 18 for IDSN = 32 primary cytoreduction (*OS* = 28) *	NACT platinum-taxol OS = 41.7 m	NACT platinum- taxol + IDSOS = 45.5 m (under 20% of patients in study)	*Choice of treatment:* upfront surgery or NACT according to disease extent and patient PPS

Main conclusions	*Upfront surgery* for advanced operable disease	Benefit of IDS versus chemotherapy alone when tumour is localised.	*NACT *for unresectable tumours leads to a group of sensitive patients for successful IDS	Response rate to NACT comparable to that of upfront surgery stated in literature	Benefit of IDS in patient responding to NACT	OS was higher for patients with high tumour load treated with NACT than with upfront surgery
	*NACT* for non operable or poor performance status with IDS ideally after 3 cycles	Best OS (48 m) with radical primary cytoreduction, TFI >24 m & ≤ 39 years		Importance of maximal secondary cytoreduction in IDS	NACT can select patients for surgery	

IDS: interval debulking surgery; m = months; NACT: neoadjuvant chemotherapy; OS: overall survival; PFS: progression free survival; PPS: patient performance status; TFI: treatment free interval.

**Table 3:** Radium hospital guidelines for IDS based on TFI and number of recurrence sites, taken from Oksefjell et al. 2009 [11].

TFI, months	Local disease	Disseminated disease
0–5	Consider SCR	No SCR
6–11	Offer SCR	No SCR
12–23	Offer SCR	No SCR
>24	Offer SCR	Consider SCR

SCR: secondary cytoreduction; TFI: treatment-free interval.
